# Rapid and quantitative detection of Shiga toxin1 and Shiga toxin2 based on multiple targets UPT‐LF assay

**DOI:** 10.1002/elsc.202000031

**Published:** 2020-07-19

**Authors:** Qiaozhen Wei, Qiushi Hu, Fengjuan Shi, Shuang Li, Chongsi Sun, Huicong Zhang, Lei Xue, QiuXia Feng, Jinying Dong, Yongjun Jiao, Lei Zhou

**Affiliations:** ^1^ National Key Laboratory of Biochemical Engineering PLA Key Laboratory of Biopharmaceutical Production & Formulation Engineering Institute of Process Engineering Chinese Academy of Sciences Beijing P. R. China; ^2^ The Department of Blood Transfusion The Second Affiliated Hospital of Anhui Medical University Hefei P. R. China; ^3^ Institute of Pathogenic Microbiology Jiangsu Provincial Center for Disease Prevention and Control Nanjing P. R. China

**Keywords:** dual‐target, lateral flow assay, *Shiga toxin*, single‐target, up‐converting phosphor technology

## Abstract

Shiga toxin‐producing *Escherichia coli* (STEC) infection causes a series of diseases that are highly pathogenic and deadly in humans and animals, seriously endangering public health. Of the pathogenic factors within STEC, the two groups of *Shiga toxin* (Stx) consisting Stx1 and Stx2 plays a prominent role in the pathogenesis of STEC infection. In this study, we developed single‐target up‐converting phosphor technology‐based lateral flow assay (Stx‐UPT‐LFA) for the rapid detection of Stx1 and Stx2, respectively, and also developed a dual‐target Stx1/2‐UPT‐LFA based on single‐target strips to detect of Stx1 and Stx2 at the meantime within 20 min. We choose the purified Stx1 and Stx2 standard samples, and the optimum monoclonal antibody (namely 8E7‐E6, 2F6‐F8 for Stx1 and S1D8, S2C4 for Stx2) were selected for use in Stx‐UPT‐LFA in double‐antibody‐sandwich mode. The sensitivities of single‐target Stx‐UPT‐LFA for both Stx1 and Stx2 were 1 ng mL^−1^ with accurate quantitation ranges of 1–1000 ng mL^−1^ and 1–800 ng mL^−1^ respectively. No false‐negative result was found in the Stx2‐UPT‐LFA even with a high‐test concentration up to 1000 ng mL^−1^. Meanwhile, both targets detection sensitivities for dual‐target Stx1/2‐UPT‐LFA were 5 ng mL^−1^, and accurate quantitation ranges were 5–1000 ng mL^−1^ and 5–800 ng mL^−1^ for standard Stx1 and Stx2 solutions without cross‐interference between two targets. Both techniques showed good linearities, with a linear fitting coefficient of determination(r) of 0.9058–0.9918. Therefore, the UPT‐LFA could realize simultaneous detection for multiple targets on a single strip and thus to quickly determine the type of infectious Stxs. In addition, the single‐target Stx1‐UPT‐LFA and Stx2‐UPT‐LFA showed excellent specificity to six toxins, even at high concentrations of 1000 ng mL^−1^. In conclusion, the developed Stx‐UPT‐LFA allows the rapid, quantitative, reliable and simultaneous detection of Stx1 and Stx2 within 20 min, providing an alternative method for clinical diagnosis of STEC infection.

**Abbreviations**
McAb: monoclonal antibody
POCT: point‐of‐care testing
STEC: *Shiga toxin*‐producing *Escherichia coli*
Stx: *Shiga toxin*
Stx1: *Shiga toxin* 1
Stx2: *Shiga toxin* 2
UCP: Up‐converting phosphors
UPT‐LFA: up‐converting phosphor technology‐based lateral flow assay

## 1 INTRODUCTION

Shiga toxin‐producing *Escherichia coli* (STEC) infection could cause severe bloody diarrhea, hemorrhagic colitis and even life‐threatening hemolytic uremic syndrome (HUS) in humans [1, 2, 3, 4]. The outbreaks and sporadic cases of STEC infection worldwide have increased in frequency over recent decade [1]. Of the pathogenic factors within STEC, Shiga toxins (Stxs) plays a prominent role in the pathogenesis of STEC infection [5]. Stxs consists of an A‐subunit monomer and a B‐subunit pentamer. A‐subunit monomer contains enzymatic RNA N‐glycosidase activity that hydrolyzes the N‐glycoside bond of adenosine of the 28S rRNA in 60S ribosomes and hence inhibits protein synthesis, while B‐subunit pentamer involves in receptor binding process [5]. Although both Stx1 and Stx2 groups of Stxs could cause human disease, epidemiological studies have revealed that Stx2‐producing strains are more frequently associated with HUS [6].

O157:H7 had been the dominant serotype of STECs since its first discovery in the United States in 1982 [7], and it could be identified through unique biochemical markers in selective and differential agars [8]. However, in recent years, Non‐O157:H7 STEC infections have been being recognized with greater frequency from an incidence of 0.12 per 100 000 population in 2000 to 0.95 in 2010 [9]. A Non‐O157 STEC, O104:H4, for instance, caused a severe food borne outbreak in Germany [10], demonstrating that Stxs not exclusively link to the certain serotype of STECs [11], and the detection of Stxs themselves (including proteins or/and encoding genes) would be a better alternative than that of their host bacteria. Additionally, the biochemical markers detection for clinical diagnosis of STEC infection is complicated and time‐consuming, which would delay the patient management and the implementation of control measures. So, developing a sensitive detection method for Stxs would complement conventional bacteria culture strategies and facilitate accurate and prompt diagnosis [12]. So far, many Stxs testing strategies including PCR for Stxs genes and immuno‐assays for Stxs proteins have been widely used clinically [13, 14, 15]. PCR is specific and sensitive, but it heavily relies on laboratory facilities and experienced personnel. Additionally, there are false‐negative results due to the PCR inhibitors in the fecal samples [14], or the total clearance of intestinal floras including STECs after excessive use of antibiotics prior to diagnosis [16]. Various enzyme immunoassays for Stxs, mainly in lateral flow (LF) and micro‐plate formats (such as colloidal gold test strip and enzyme‐linked immunosorbent assay [ELISA], etc), are commercially available [13, 15]. However, some of them suffer from poor sensitivity due to the low concentration in broth culture [12, 17, 18]. At present, point‐of‐care testing (POCT) plays a key role in early diagnosis and screening for disease. Therefore, it is urgent to develop a rapid and easily performed assay to facilitate detection of Stxs. And an inexpensive, convenient, and reliable POCT is especially essential for resource‐limited areas.

In recent years, with the rapid development of in vitro diagnostic technology, it's an ongoing challenge to search for simple and inexpensive assays with higher detection sensitivity and the more accurate quantitative capability [19, 20]. In this respect, LF immunoassay, also known as immunochromatographic analysis is widely acknowledged for that they are not only low cost, ease‐to‐use, highly sensitive but also well suited for rapid on‐site testing [21]. In addition, using colloidal gold or fluorescent microsphere as reporter in immunochromatography was the classical POCT technique [22, 23], which were widely used in biomedical field since 1990s. However, these methods can only be used for qualitative or semi‐quantitative detection, which greatly limits its wide application in STEC infection fields. Thus, a series of LF assays based on immunochromatography with new labels, such as magnetic nanobeads, quantum dots and up‐converting phosphor, have attracted more and more attention nowadays [24, 25, 26]. POCT for rapid, sensitive and selective detection of molecular biomarkers have developed rapid, especially up‐converting nanoparticles and other luminescent materials as the reporter have attracted more and more researchers' interest [27, 28, 29]. Compared with the other type of LF assays, the up‐converting phosphor technology‐based lateral flow assay (UPT‐LFA) is a new emerging type of LF assays with the unique optical features of the anti‐Stokes shift and the stable fluorescence of Up‐converting phosphors (UCP) [30], which allows for the quantitative detection of pathogens by non‐technical personnel [31, 32]. It mainly has been used in drugs abuse detection and pregnancy testing at the early stage [33]. Now, it has been surging in infectious disease, pathogens and tumors diagnostics [34, 35, 36].

In UPT‐LFA, UCP, as a new biological tracer, are particles (∼50 nm) composed of rare earth lanthanide elements embedded in a crystal with the unique property of absorbing two or more photons of low‐energy infrared light to emit a single, higher‐energy, visible photon [37]. This property has not found in natural biological materials, UCP particles are no background autophosphorescence from the carrier fluid or the assay bio‐chemistry. The only problem for background should be concerned is nonspecific binding of the phosphors to the capture site [38]. Compared to other fluorescent labels including fluorescein isothiocyanates, gold nanoparticles, carbon quantum dots [39, 40, 41], UCP particles have strong chemical inertness and no optical attenuation. They do not react with conventional biochemical materials and are not subject to sample corrosion or the decay of the marker itself. They have durable light‐emitting characteristics and can be kept for long periods and repeatedly tested to ensure the stability and safety of the technology in the detection process [42]. Besides, based on double‐antibody sandwich assay, the use of high‐affinity monoclonal antibodies can guarantee sufficient specificity and avoid of interference of complex components in the sample of the assay, thus providing a solid foundation for the rapid and highly sensitive detection of pathogens.

Up until now, as a result of work by our research team on covalent coupling between UCP and antibodies, as well as quantization of reaction signals through UPT biosensors, several kinds of UPT‐LFA have been developed for quantitative detection of pathogens, including drugs, nucleic acids, cytokine, antibodies, and pathogens [32, 43, 44, 45, 46].

In this study, specific antibodies were covalently bounded to the UCP particles to establish the UPT‐LFA for Stx1 and Stx2. We developed Stx‐UPT‐LFA including two patterns for single‐ and dual‐target rapid quantitative detection of Stx1, Stx2 and evaluated its performance. Stx‐UPT‐LFA provided an alternative method to undertake POCT to facilitate detection of STEC infection due to its excellent sensitivity, specificity, easy operation, less sample and time‐consuming.

**PRACTICAL APPLICATION**

In this work, we developed three types of *Shiga toxin* (Stx) up‐converting phosphor technology‐based lateral flow assay (Stx‐UPT‐LFA) for the rapid detection of Stx1 and Stx2, respectively, and also detection of Stx1 and Stx2 at the meantime within 20 min. Different from previous UCP‐LFA, to our knowledge, this is the first application and evaluation of UPT‐LFA in detection of Stx1 and Stx2 on a single strip and without cross‐interference. Although one‐step multi‐target detection in endemic regions is not a confirmatory test to diagnose STEC infection, it gains potential when the type of different classes of toxin specific for the STEC infection can be determined. In a word, owing to its rapidness, ease of operation, good performance, and low cost, after comprehensive evaluation, we believe that the Stx‐UPT‐LFA should be considered as a promising approach for the rapid detection of Stx1 and Stx2 in clinical diagnosis of STEC infection.

## 2 MATERIALS AND METHODS

### 2.1 Reagents and instruments

UCP (NaYF4:Yb^3+^, Er^3+^) nanoparticles with a size of ∼ 50 nm [47] was provided by Dr. Yan Zheng from Shanghai Kerune Phosphor Technology Co., Ltd. (Shanghai, China). UPT‐based biosensor was developed through cooperating with Shanghai Institute of Optics and Fine Mechanics, Chinese Academy of Sciences (Shanghai, China) [48]. Nitrocellulose membrane (SHE 1350225) and glass fiber (GFCP20300) were purchased from Millipore Corp. (Bedford, MA, USA). Absorbent papers (Nos 470 and 903) were purchased from Schleicher & Schuell, Inc. (Keene, NH, USA). Plastic cartridges for strips were designed by our group and produced by Shenzhen Jincanhua Industry Co. (Shenzhen, China).

Tetraethyl orthosilicate, 3‐aminopropyltriethoxysilane, polyoxyethylene, glutaraldehyde, BSA, NaN3 and Triton NP‐40 was purchased from Sigma‐Aldrich (St. Louis, MO, USA). Other reagents are analytical pure and purchased from Sinopharm Chemical Reagent Co., Ltd. (Shanghai, China).

Standard toxins of Stx1 and Stx2, different monoclonal antibody (McAb) pairs against Stx1 and Stx2 (namely 8E7−E6 and 2F6−F8 of Stx1, S2C4 and S1D8 of Stx2) were obtained from Key Laboratory of Enteric Pathogenic Microbiology, Ministry of Health, Jiangsu Provincial Center for Disease Prevention and Control (Nanjing, China). AflatoxinB1, AflatoxinM1, Ricin, Arbin, Ochratoxin were purchased from Huaan Magnech Bio‐Tech Co. Ltd (Beijing, China). In addition, goat anti‐mouse IgG, rabbit anti‐goat IgG and goat IgG were prepared and purified by our research team.

### 2.2 Conjugation of Stx McAb to UCP

UCP particles were coated with a thin layer of silica by using tetraethyl orthosilicate. Then, the silica‐coated surface of UCP can be functionalized with amino‐, aldehyde‐functional groups using 3‐aminopropyltriethoxysilane, polyoxyethylene bis‐amino and glutaraldehyde, respectively. These aldehyde‐functionalized UCP can covalently conjugate with anti‐Stx‐mAbs (namely 2F6‐F8 or S2C4) directly in a 4°C‐prechilled Na_2_CO_3_–NaHCO_3_ buffer (50 mM, pH 9.5) under stirring. Uncoupled antigen to UCP particles was separated from the conjugated ones by a series of washing steps involving centrifugation and resuspension of pelleted UCP in 30 mM phosphate buffer (pH 7.2). After several round of washes, UCP‐Stx‐McAb conjugates (1 mg mL^−1^) were stored at 4°C in 30 mM phosphate buffer [pH 7.2, containing 0.1% w/v BSA, 0.02% w/v NaN_3_] for future use.

### 2.3 Preparation of Stx‐UPT‐LF strips

#### 2.3.1 Single‐and dual‐target strips development

**FIGURE 1:**
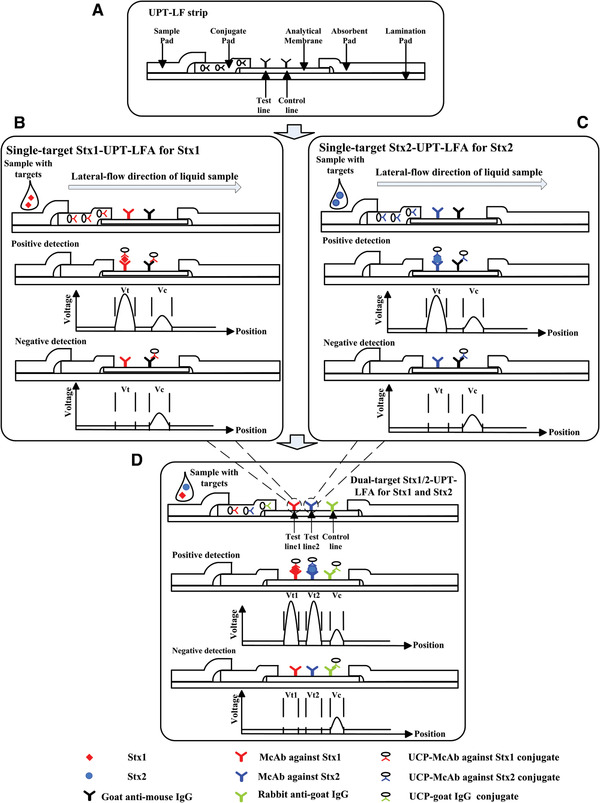
Schematic diagram of the structure and detection results of the up‐conversing particle‐based lateral flow assay (UPT‐LFA). (A) UPT‐LFA strip with a similar structure to that of a traditional gold colloid‐based lateral‐flow strip. The sample pad, conjugate pad, analytical membrane, and absorbent pad were mounted on a laminated card with proper overlaps. All biomolecules for specific detection were fixed in advance, including the UCP−antibody conjugate in the conjugate pad and antibodies for test and control lines on the analytical membrane. For (B) Stx1 (C) Stx2 detection, a pair of specific antibodies for the target were used to conjugate with UCPs and as the test line, respectively. During positive detection, the captured target formed a UCP−antibody−target−antibody complex on the test line and then produced a detectable, quantifiable optical signal that depended on the relationship between the ratio of the peak areas of the test and control lines (T/C) and the target concentration in the sample. (D) For dual‐target detection of Stx1 and Stx2, two test lines were included with each corresponding to a certain target, allowing two targets to be qualitatively and quantitatively detected during a single operation

UPT‐LF strips for single‐target detection of Stx1 or Stx2 were firstly produced by imitating the construction of traditional gold colloid‐based lateral flow strips (Figure 1A), and were named Stx1‐UPT‐LFA strip and Stx2‐UPT‐LFA strip, respectively. Before assembling Stx‐UPT‐LF strip, several steps had to be taken. First, UCP–Stx1‐2F6‐F8 or UCP‐Stx2‐S2C4 conjugate was diluted to 0.5 mg mL^−1^ by conjugate‐diluting phosphate buffer (10 mmol L^−1^, pH 7.2, containing 1% w/v BSA and 0.5% v/v Triton X‐100) before being added onto the conjugate pad at 0.1 mL cm^−1^, and then the pad was dried at 37°C for 3 h. Second, another anti‐Stx‐McAbs (namely 8E7‐E6 and S1D8, 2 mg mL^−1^) and goat anti‐mouse IgG (2 mg mL^−1^) were loaded on a nitrocellulose membrane at 1 μL cm^−1^ as the test line (T) and control line (C), respectively, and dried at 37°C for 1 h for use as a analytical membrane (see Figure 1B,C). Finally, to assemble LF strips, the analytical membrane, the conjugate pad, the sample pad, and the absorbent pad were pasted on the lamination card with proper overlapping sequence. The test strip was cut into 4 mm in width with the guillotine cutter (CM4000, BioDot) and put into the plastic case. Dual‐target UPT‐LF strips for simultaneous detection of Stx1 and Stx2 were also fabricated in a similar manner and denoted Stx1/2‐UPT‐LFA. To prepare this conjugate pad, UCP−Stx1‐McAb, UCP−Stx2‐McAb, and UCP−goat IgG conjugates were mixed together with concentrations of 1.5 mg mL^−1^. Another anti‐Stx McAbs and rabbit anti‐goat IgG with concentrations of 2 mg mL^−1^ were dispensed on a nitrocellulose membrane as the test line 1 (T1), test line 2 (T2), and the control line (C), respectively (Figure 1D).

#### 2.3.2 Development and optimization of the Stx‐UPT‐LFA

Standard Stx1 or Stx2 solutions (10 μL) was mixed with sample treating buffer (90 μL, 0.03 M phosphate buffer that contained 1% BSA, 0.5% SDS and 0.25 M NaCl, pH 7.2), and added dropwise onto a strip subsequently, which was analyzed by the UPT‐based biosensor after standing 15 min to allow sample flow and immunoreaction. In positive detection, the detected target first combines with a UCP−antibody conjugate located in the conjugate pad and then captured by another pairing antibody immobilized as the T line on the analytical membrane during LF. This immobilizes the UCPs with the detected target as a bridge to give a detectable, quantifiable signal corresponding to the target concentration in the sample. In negative detection, because of the absence of the target, UCPs are only captured by the C line, so the optical signal of UCPs is detected on the C line but not the T line. The peak areas corresponding to the T and C lines that the ratio of T to C (T/C) is used to judge the concentration of the target in the tested sample qualitatively and quantitatively. By including two T lines, dual targets could be detected simultaneously (Figure 1D).

#### 2.3.3 Evaluation of sensitivity, linearity and precision for the single‐target Stx‐UPT‐LFA

A standard toxin of Stx1 or Stx2 was serially diluted with sample treating buffer to give concentrations from 0,1,5,10,20,40,60,80,100,200,400,600,800,1000 ng mL^−1^ and then detected by Stx1‐UPT‐LFA or Stx2‐UPT‐LFA, respectively. The cutoff threshold was determined using LB broth as the negative sample and equaled the mean of T/C plus three times the standard deviation (Mean _T/C_ + 3SD). Samples with T/C larger than cutoff threshold were positive and vice versa. The lowest concentration that gave T/C larger than the cutoff threshold was determined as the limit of detection, which represents the sensitivity of the assay, and the correlation coefficient of linear regression was obtained to assess the accuracy of quantification. The coefficient of variation for three trials was used to evaluate the precision of the strips.

#### 2.3.4 Evaluation of specificity for the single‐target Stx‐UPT‐LFA

Five common toxins including ricin, aflatoxin B1, aflatoxin M1, ochratoxin and abrin, were used for specificity evaluation. Each toxin was diluted to 1000 ng mL^−1^ using sample‐treating buffer, and then detected by Stx1‐UPT‐LFA and Stx2‐UPT‐LFA, respectively. Finally, standard toxins (Stx1 and Stx2) at 1,10,100,1000 ng mL^−1^ were also used to evaluate the specificity of single‐target Stx1‐UPT‐LFA and Stx2‐UPT‐LFA, respectively.

#### 2.3.5 Simultaneous detection by a dual‐target Stx1/2‐UPT‐LFA

The standard toxins of Stx1 and Stx2 were diluted to 0, 5, 10, 40, 80, 100, 400, 800, 1000 ng mL^−1^ using sample‐treating buffer, respectively. All samples were tested using dual‐target Stx1/2‐UPT‐LFA three times. Under nontarget interference, if the T/C ratio of the negative sample was still lower than the cutoff value, then it means that the dual‐target UPT‐LFA shows good anti‐cross‐interference performance for simultaneously detected targets for Stx1 and Stx2.

## 3 RESULTS AND DISCUSSION

### 3.1 Sensitivity, linearity and precision of single‐target Stx‐UPT‐LFA

**FIGURE 2:**
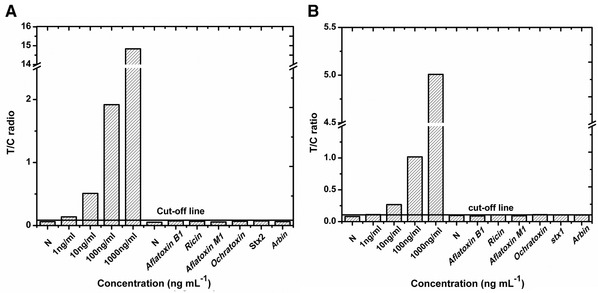
Specificity assessments of single‐target Stx1‐UPT‐LFA and Stx2‐UPT‐LFA. The single‐target Stx1‐UPT‐LFA and Stx2‐UPT‐LFA showed excellent specificity of six toxins as potential bioterrorism agents and have a similar structure or a similar contamination routes to Stx1 and Stx2

**FIGURE 3:**
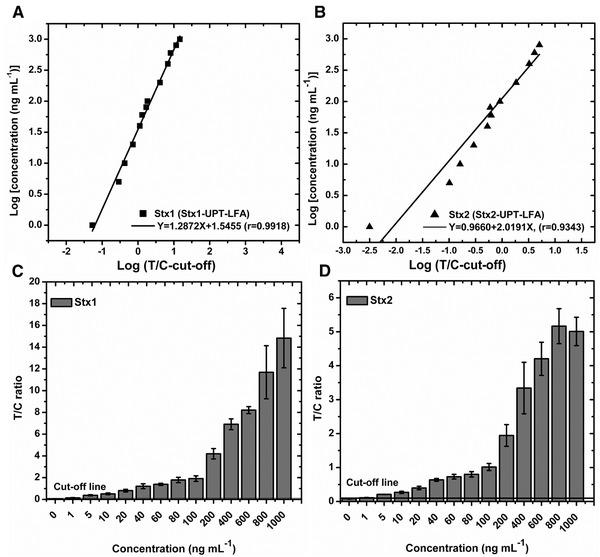
Standard quantification curve of single‐target Stx‐UPT‐LFA obtained by Stx1 and Stx2 diluted by sample treating buffer. The sensitivities of single‐target Stx1‐UPT‐LFA and Stx2‐UPT‐LFA were all 1 ng mL^−1^ with accurate quantitation of 1–1000 ng mL^−1^ and 1–800 ng mL^−1^, the Stx2‐UPT‐LFA haven't appear false‐negative results even at high concentrations of 1000 ng mL^−1^

After systematic optimizations, the performance of the assay was evaluated by testing a series of standard Stx1 or Stx2 solutions ranging in concentration from 1 to 1000 ng mL^−1^. The cut‐off threshold for the Stx‐UPT‐LFA was determined by testing the blank samples (LB) (mean _T/C_ + 3SD); samples with T/C ratio higher than the cut‐off value were determined as positive and vice versa. The sensitivities of the Stx1‐UPT‐LFA and Stx2‐UPT‐LFA all were 1 ng mL^−1^. A standard curve and the quantitative equation are shown in Figure 2, with the logarithm of the T/C–cut‐off value as x and the logarithm of the Stx concentration assay. The correlation coefficients of linear regression analysis for the standard quantitation curve were all above 0.9, (*p* < 0.0001), demonstrating good linearity for the quantification of Stx1 and Stx2 in a wide concentration range of 1–1000 ng mL^−1^ and 1–800 ng mL^−1^ except the T/C ratio decreased slightly of Stx2 at 1000 ng mL^−1^ (Figure 3B,C). The possible reason was that high toxin concentrations maybe clogged the pores on the strips and blocked the reactions among antibodies and antigens. The signals at the T line declined more obviously than those at the C line because the T line was closer to the sample, thereby leading to a decrease in the T/C ratio at high toxin concentrations, the Stx2‐UPT‐LFA haven't appear false‐negative results, demonstrating that the precision of single‐target Stx‐UPT‐LFA was suitable for POCT use.

### 3.2 Specificity of single‐target Stx‐UPT‐LFA

**FIGURE 4:**
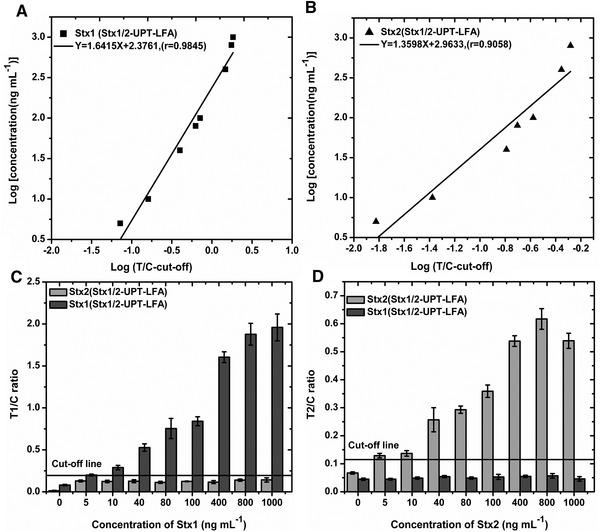
Standard quantification curve of dual‐target Stx1/2‐UPT‐LFA obtained by Stx1 and Stx2 diluted by sample treating buffer. the sensitivities of dual‐target Stx1/2‐UPT‐LFA all were 5 ng mL^−1^ with accurate quantitation of 5–1000 ng mL^−1^ and 5–800 ng mL^−1^ for standard Stx1 and Stx2 solutions without cross‐interference between two targets

Toxins that have a similar structure or a similar contamination route to those of Stxs or that could potentially be used as bioterrorism agents were selected to evaluate the specificity of the assay. These included ricin, aflatoxin B1, aflatoxin M1, ochratoxin, Abrin, Stx1 and Stx2. None of these toxins were detected out by the Stx1‐UPT‐LFA and Stx2‐UPT‐LFA (Figure 4A,B), even at high concentrations of 1000 ng mL^−1^. These results demonstrate the high specificity of the assay for the detection of Stx1 or Stx2.

### 3.3 Evaluation of dual‐target Stx1/2‐UPT‐LFA

Due to we systematically evaluated the specificity of the single‐target Stx‐UPT‐LFA for toxins Stx1 and Stx2, the two most important properties of multiple detection are sensitivity and anti‐interference in the presence of the detected target at high concentration. Therefore, a series of Stx1 and Stx2 solutions were detected using Stx1/2‐UPT‐LFA. Here, T1/C represents the detection result for Stx1, and T2/C represents that for Stx2. The sensitivities of Stx1/2‐UPT‐LFA for detection of both Stx1 and Stx2 were 5 ng mL^−1^, which was 5‐fold lower than that of Stx1‐UPT‐LFA and Stx2‐UPT‐LFA.Though the sensitivity of Stx1/2‐UPT‐LFA for Stx1 and Stx2 were poorer than the single‐target Stx‐UPT‐LFA, the correlation coefficient (r) of linear correlation were determined to be 0.9845 and 0.9058, demonstrating good linearity for the quantification of Stx1 and Stx2 in a wide concentration range of 5–1000 ng mL^−1^ and 5–800 ng mL^−1^. Furthermore, non‐target toxins results were lower than the cut‐off threshold when detected target toxins by the Stx1/2‐UPT‐LFA, even at high concentrations of 1000 ng mL^−1^. Thus, Stx1/2‐UPT‐LFA showed an excellent anti‐interference ability (Figure 4C,D). Due to this reason, compared to the single target detection, specific toxin types can be quickly detected in a relatively short time by the dual‐target Stx1/2‐UPT‐LFA, which helps to early prevention and treatment to some extent.

## 4 CONCLUSION

In this study, we successfully developed a rapid and easily performed single‐target Stx‐UPT‐LFA and dual‐target Stx1/2‐UPT‐LFA to facilitate detection of Stxs and thus to meet the different requirements of practical application. In single target detection, the sensitivities of Stx1 and Stx2 were 1 ng mL^−1^ for both Stx1‐UPT‐LFA and Stx2‐UPT‐LFA, attributable to the use of more sensitive reporter, UCPs, and the high‐affinity anti‐Stx‐McAb prepared in this study. Stx1 and Stx2 can also be quantitated in a wide concentration range of 1–1000 ng mL^−1^ and 1–800 ng mL^−1^ (*r* = 0.9918 and *r* = 0.9343). No significant cross‐reactivity was observed with several other toxins. Moreover, though the dual‐target Stx1/2‐UPT‐LFA sensitivities of Stx1 and Stx2 were 5 ng mL^−1^ poorer than the single‐target Stx‐UPT‐LFA, the assay showed good linearity, with a linear fitting coefficient of determination (*r*) of 0.9845 and 0.9058 for standard Stx1 and Stx2 solutions without cross‐interference between two targets, and could finish the test within 20 min, which provides a reliable basis for simultaneous detection of multiple targets. However, due to the lack of precise sample pretreatment, the complex components in the sample lead to a certain fluctuation in the UPT‐LFA results. Therefore, UPT‐LFA was usually used as a rapid screening method, the suspected samples still need to be further validated by the reference method in the lab. Although one‐step multi‐targets detection in endemic regions is not a confirmatory test to diagnose STEC infection, it is still a potential method due to the different type toxin specific for the STEC infection could be determined. In a word, owing to its rapidness, ease of operation, good performance, and low cost, dual‐target detection is adapted to the development of POCT detection recently. Therefore, after comprehensive evaluation, we believe that the single‐ and dual‐target Stx‐UPT‐LFA should be considered as a promising approach for the rapid detection of Stx1 and Stx2 in clinical diagnosis of STEC infection.

## CONFLICT OF INTEREST

The authors have declared no conflict of interest.
